# BirdNET can be as good as experts for acoustic bird monitoring in a European city

**DOI:** 10.1371/journal.pone.0330836

**Published:** 2025-09-11

**Authors:** Andrew J. Fairbairn, Josija-Simeon Burmeister, Wolfgang W. Weisser, Sebastian T. Meyer

**Affiliations:** 1 Terrestrial Ecology Research Group, Department of Life Science Systems, School of Life Sciences, Technical University of Munich, Freising, Germany; 2 Faculty of Forest Sciences and Forest Ecology, Georg-August University of Göttingen, Göttingen, Germany; Lyon College, UNITED STATES OF AMERICA

## Abstract

BirdNET has become a leading tool for recognising bird species in audio recordings. However, its applicability in ecological research has been questioned over the sometimes large number of species falsely identified. Using species-specific confidence thresholds has been identified as a powerful approach to solving this issue. However, determining these thresholds is time and resource-consuming. While optimising the parameter setting of the algorithm could be an alternative strategy, the effect of parameter settings on the algorithm’s performance is not well understood. Here, we compared the species identification of BirdNET against expert identification using an acoustic dataset from a single site in Munich, Germany. The performance of BirdNET was evaluated using three performance metrics: precision, recall, and F1-score, using 24 combinations of the parameters: week, sensitivity, and overlap at four temporal aggregations (pooling of data across time intervals). We found that BirdNET performance varied widely depending on parameter settings (0.46–0.84). When given more data (higher temporal aggregation) and with tuned parameters, BirdNET came close to matching the expert identification (F1 score = 0.84). While BirdNET missed five species of the 23 species identified by the experts, our confirmation test revealed that BirdNET also found one species missed by the experts. To understand how each parameter affects F1 score, we trained linear mixed effects models. Our models showed that the confidence threshold had the strongest effect on the F1 score (p < 0.001) and significantly interacted with temporal aggregation, sensitivity, and overlap. Our results showed that while there are still limitations, using appropriate parameter settings, aggregating results over longer periods and undertaking some basic validation, BirdNET can yield results comparable to experts without the need for time-consuming estimation of species-specific thresholds.

## Introduction

Monitoring bird communities traditionally relies on field observations, requiring experts to manually identify species through visual or auditory cues. While effective, this process is time-intensive, subject to observer bias [1,2], and limited in spatial and temporal coverage [3]. Passive acoustic monitoring (PAM) has emerged as an alternative, allowing continuous data collection across multiple sites simultaneously. However, the bottleneck in analysing the vast amounts of audio data generated through PAM has limited its practical application in ecological research, as manual species identification in recordings remains equally time-consuming and requires specialised expertise [4].

Recent advancements in machine learning have transformed acoustic data analysis, making automated species identification increasingly accessible to researchers without computer science expertise. Among these tools, BirdNET [5] has emerged as a leading tool. The current version, 2.4, has global coverage of over 6,500 avian species as well as other classes of (non-avian) sound [6]. In Germany, BirdNET covers 407 of the 527 bird species tracked by the German Ornithologists’ Society, including rare and vagrant species [7]. This extensive coverage, coupled with its open-source nature and user-friendly interface, has driven BirdNET’s rapid adoption in both industry applications and scientific research [e.g., 8–10].

Despite its growing popularity, integrating BirdNET in ecological research has been questioned over the sometimes large number of species falsely identified. Using species-specific confidence thresholds has been identified as a powerful approach to solving this issue [6]. As such, many BirdNET studies have focused on optimising confidence thresholds [11,12]. However, determining these thresholds is time and resource-consuming. Optimising the parameter setting of the algorithm could be an alternative strategy to improve suboptimal classifications and increase the reliability of ecological metrics derived from BirdNET analyses. Yet the effect of parameter settings—such as overlap, sensitivity, and week of the year—on BirdNET’s performance is not well understood [9], especially in an urban environment.

Here, we used expertly identified acoustic recordings collected in Munich, Germany, to test BirdNET for bird species classification. We aim to determine whether BirdNET can provide species lists comparable to an expert ornithologist in an urban environment. More specifically, we a) assess the impact of varying BirdNET parameters on classification performance, b) examine how different temporal aggregations influence output, and c) compare BirdNET’s performance to expert annotations in terms of species richness and identification accuracy. Based on these findings, we provide practical recommendations for parameter settings and validation approaches that optimise BirdNET performance in urban acoustic surveys.

## Methods

### Acoustic recording

We placed a single Frontier Labs BAR on the roof of a housing complex in Munich, Laim, between June and October 2021. We recorded in week-long blocks, with a minimum of one week between recording periods. We recorded one minute every 10 minutes from two hours before sunrise to three hours after sunrise [13], to keep the amount of manual identification required to a manageable level, resulting in a total of 15.5 hours of recordings over 60 days. Recordings were taken at a sample rate of 48 kHz, a bit depth of 16 and a gain of 40 dB.

### Species identification

Two experts (A.J. Fairbairn, J.S. Burmeister) identified all bird vocalisations in each recording visually or aurally using Kaleidoscope Pro version 5.6.8 [14] to view the spectrograms and listen to the recordings, resulting in a list of species for each one-minute recording. The default parameters were used (FFT size: 256, Window size: 128, Max cache size: 256 MB). Next, we ran BirdNET-Analyzer (April 2023) model version v2.2 on the same recordings, producing a list of BirdNET species detections for each one-minute recording.

### Analysis

#### Parameter effects.

We tested four parameter settings. First, BirdNET can use the *week* of the year of recording in conjunction with the location to filter what species are likely to occur at a location at that time of the year, using eBird [15] species lists. We ran all analyses with and without week included. Second, the detection *sensitivity* (range 0.5 (low) to 1.5 (high), default 1.0) affects how sensitive BirdNET is to faint or background vocalisations. We ran all analyses with three sensitivity levels (0.5, 1.0, 1.5). Third, BirdNET works on three-second audio segments for analysis. The *overlap* determines how many seconds of the previous segment are “overlapped” (default 0.0s). We ran all analyses with four levels of overlap (0, 1, 2, 2.9 seconds). Finally, BirdNET provides a *confidence* threshold, i.e., the confidence that BirdNET has in its own predictions [6], for each detection. Setting a minimum confidence threshold in BirdNET causes all detections with a lower confidence to be removed from the results. Thus, to identify the best settings, including the best minimum confidence threshold, we ran all analyses with the default minimum confidence threshold (0.1) to get a full list of detections that we could filter afterwards for the analyses using steps of 0.01.

#### Temporal aggregation.

To test how different temporal aggregation (i.e., short versus long recording periods) affect BirdNET’s performance, we aggregated both our reference data and BirdNET results to four different temporal scales: minute (no aggregation), day, week, and the entire dataset (Table 1). For BirdNET results, we first filtered the raw detections based on confidence thresholds (steps of 0.01) and the other parameters (week, overlap, sensitivity) before aggregation. For each temporal scale, we recorded only species presence data, ignoring repeated detections of the same species. At the minute level, if a species was detected multiple times within the same minute, it was recorded as a single presence. At the day level, if a species was detected in any minute during that day, it was recorded as a single presence for the entire day. At the week level, any species detected at any point during the week was recorded as a single presence for that week. For the entire dataset, each species was recorded as either present or absent overall. This presence-based aggregation was applied to both the expert identifications and filtered BirdNET outputs before making any comparisons.

**Table 1:** Breakdown of how the data was aggregated to get the different temporal aggregation levels.

level	length	total comparisons
minute	1 minute	930
day	31 minutes	30
week	155 minutes*	5
dataset	930 minutes	1

* Two weeks varied slightly by the number of recordings. 155 minutes is the average.

#### BirdNET vs expert.

To compare BirdNET’s output to expert identifications, we calculated three metrics for each aggregation level: true positives, defined as species correctly detected as present by BirdNET; false positives, defined as species incorrectly reported as present by BirdNET but not confirmed by experts; and false negatives, defined as species confirmed present by experts but not detected by BirdNET . We did not calculate true negatives, as there is no meaningful “absence” class in this context—only species that may have been missed by either the expert or BirdNET. Based on these values, we calculated three commonly used machine learning evaluation metrics: precision, recall, and F1 score, to assess BirdNET’s performance. Precision answers the question “How reliable are BirdNET’s identifications?” by measuring the proportion of BirdNET’s species detections that were correct (Eq. 1). A high precision means that when BirdNET identifies a species, it’s likely to be accurate. Recall answers the question “How comprehensive is BirdNET’s coverage?” by measuring the proportion of actually present species (as identified by experts) that BirdNET successfully detected (Eq. 2). A high recall means BirdNET is capturing most of the species present in recordings. Because there is typically a trade-off between precision and recall—improving one may reduce the other—we use the F1 score (Eq. 3) as a balanced performance metric. The F1 score represents the harmonic mean of precision and recall, providing a single value that is high only when both precision and recall are high. This offers an integrated measure of BirdNET’s overall effectiveness at correctly identifying bird species while minimising both false identifications and missed species. Importantly, these metrics were calculated at each aggregation level, meaning we compared the complete species list for each time unit (minute, day, week, or entire dataset) generated by BirdNET to that identified by the expert rather than evaluating individual detections.

To identify the best parameter settings for each aggregation level, we selected the settings that provided the highest F1 score. Where multiple parameter settings yielded the same F1 score, we selected the settings closest to default. If the settings were the same (e.g., same setting but different minimum confidence), we selected the settings with the highest minimum confidence. Using the settings that produced the best overall F1 scores, we then calculated separate F1 scores for each individual minute, day, and week to assess how performance variability changes across different temporal aggregation levels.


$$Precision=\frac{True\;Positive}{True\;Positive+False\;Positive}$$ (1)

$$Recall=\frac{True\;Positive}{True\;Positive+False\;Negative}$$ (2)

$$F1=2\times \frac{Precision\times Recall}{Precision+Recall}$$ (3)

#### Test of parameter settings on BirdNET performance.

To understand the effects of each parameter, we fitted a linear mixed-effects model with F1 score as the response variable. Fixed effects included the main effects of aggregation level, sensitivity, overlap, and minimum confidence (confthresh²), as well as all pairwise interactions among them. A random intercept was included for each run to account for repeated runs of BirdNET. Models were fitted using the *lme* function from the *nlme* package [16] in R [17] (Eq 4; S1 Table in S1 Text).


$$\begin{array}{c} \begin{array}{c} f1\;score\;\sim \;level+sensitivity+overlap+confthresh²\\ \;\;\;\;\;\;\;\;+\;level:sensitivity+level:overlap+level:\;confthresh²\\ \;\;\;\;\;\;\;\;+sensitivity:overlap+sensitivity:confthresh²\\ \;\;\;\;\;\;\;\;+overlap:confthresh²,\;random=1|run \end{array} \end{array}$$ (4)

#### Confirmation test.

While in the previous steps, we assumed that the expert identification was perfect, incorrect identification or missed species may also occur in the species lists based on expert identification, despite our best efforts. Therefore, we conducted a confirmation test assuming errors can occur within expert and BirdNET identification. Using the parameter values that produced the best result for the dataset level (highest F1 score), we manually checked a portion of the BirdNET detections. Following Sethi et. al. 2021 [8], we sorted the BirdNET results by species and randomly selected up to 50 results for each species. For species with fewer than 50 detections, we reviewed all available detections. Each selected detection was re-examined by listening to the audio and confirming whether it was correctly identified. We additionally examined the confidence ranges of any false negative species (species identified by the experts but missed by BirdNET when using the best parameter settings) to determine what the impact of lowering the confidence threshold would be.

## Results

We recorded 930 minutes over five weeks and expertly identified 9466 vocalisations of a total of 23 species in the recordings. The most commonly identified species were Short-toed Treecreeper *Certhia brachydactyla* (n = 2894), Blackbird *Turdus merula* (n = 1078), Common Swift *Apus apus* (n = 817) and Great Tit *Parus major* (n = 798; S2 Table in S1 Text). With default settings (overlap 0s, sensitivity 1, confidence 0.1), and including week of the year, BirdNET detected 13787 vocalisations from 93 species with the most frequently identified being the European Robin *Erithacus rubecula* (n = 2890), the Blackbird (n = 1820), the Great Tit (n = 1264), and the Short-toed Treecreeper (n = 1037; S3 Table in S1 Text). With respect to BirdNET’s most frequently identified species, experts identified the European Robin as the fifth most common species with 527 vocalisations. Of BirdNET’s 2890 Robin detections, 63% had confidence scores ≤0.3.

### BirdNET vs expert

**Fig 1:**
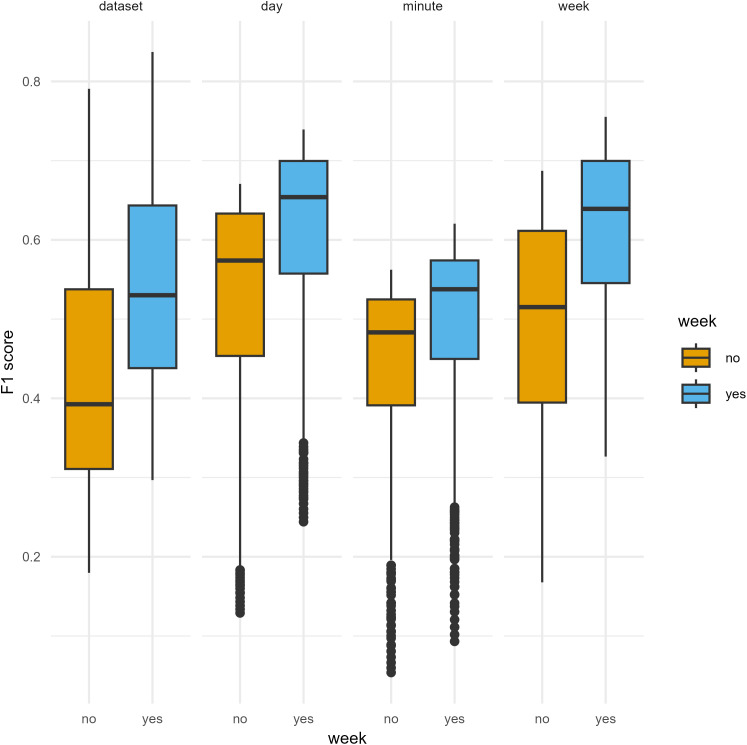
F1 scores measuring BirdNET accuracy compared to expert identification when including or not including week of the year, tested across four temporal aggregation levels with various parameter settings.

**Fig 2:**
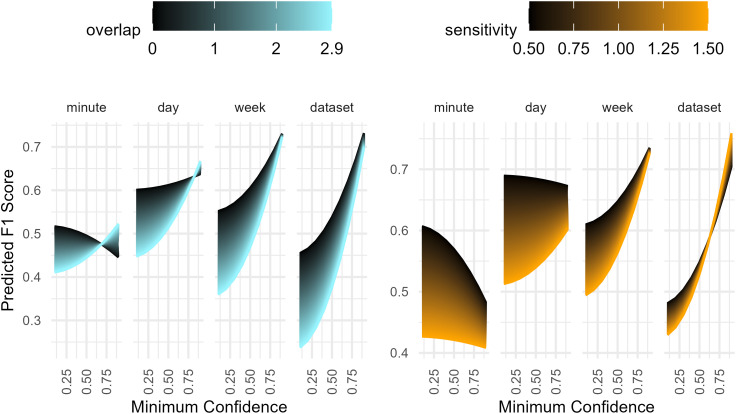
Predicted F1 score across temporal aggregation levels and BirdNET parameters. Predicted F1 score as a function of temporal aggregation level, overlap, sensitivity, and minimum confidence from a linear mixed-effects model based on 1,944 BirdNET parameter configurations, each tested against expert identifications within four aggregation levels (7,776 comparisons total). Each panel shows variation in predicted F1 scores across aggregation level, minimum confidence, and overlap (left) or sensitivity (right).

When comparing BirdNET with expert identification, temporal aggregation and all tested parameters (minimum confidence, week, sensitivity, and overlap) significantly affected BirdNET performance, which varied substantially (Range of F1 scores: 0.054 to 0.837 (mean ± SD = 0.519 ± 0.138); Figs 1, 2, Tables 2 and S4 in S1 Text). Including the *week* of the year consistently provided better results (Fig 1). Our linear mixed effects models showed that confidence threshold had the strongest effect on F1 score (p < 0.001) and significantly interacted with temporal aggregation, sensitivity, and overlap (Fig 2, S1 Table in S1 Text). As aggregation increased from the minute level to the whole dataset, maximum observed F1 scores improved from 0.62 to 0.84, respectively, when using optimal combinations of minimum confidence, sensitivity, and overlap (i.e., those providing the best F1 scores, Table 3). With the exception of high confidence thresholds for the minute and day aggregations, decreasing overlap provided higher maximum F1 scores (Fig 2, S4 Table in S1 Text). Default BirdNET parameter settings performed poorly when considering F1 scores (Table 3). However, default settings provided higher recall, maximising the number of true positives while inflating false positives. Interestingly, our models revealed that the effect of overlap flips with increasing confidence threshold, showing that at the minute and day levels, a high confidence and overlap provided the best F1 scores while sensitivity had the opposite effect (Fig 2).

**Table 2:** Summary of BirdNET performance (F1 score) for each parameter, averaged across all combinations of the other parameters and aggregation levels. Values represent the range and mean ± standard deviation of observed F1 scores.

parameter	value	range	mean_sd
aggregation	minute	0.054-0.62	0.471 ± 0.111
	day	0.129-0.739	0.571 ± 0.129
	week	0.168-0.755	0.554 ± 0.132
	dataset	0.18-0.837	0.481 ± 0.148
overlap	0	0.124-0.837	0.551 ± 0.127
	1	0.106-0.818	0.552 ± 0.129
	2	0.088-0.829	0.528 ± 0.132
	2.9	0.054-0.818	0.446 ± 0.135
sensitivity	0.5	0.235-0.755	0.546 ± 0.113
	1	0.18-0.755	0.531 ± 0.123
	1.5	0.054-0.837	0.48 ± 0.163
week	no	0.054-0.791	0.472 ± 0.135
	yes	0.093-0.837	0.566 ± 0.124

**Table 3:** Best-performing BirdNET parameter settings resulting in the highest F1 scores within each temporal aggregation level, based on 1,944 model configurations compared to expert identifications per temporal aggregation level (7,776 comparisons total).

aggregation	week	sensitivity	overlap	minConf	precision	recall	F1 score	true positives	false positives	false negatives
minute*	yes	1	0	0.1	0.35	0.69	0.46	1196	2267	546
**minute**	**yes**	**1**	**2**	**0.54**	**0.70**	**0.56**	**0.62**	**975**	**426**	**767**
day*	yes	1	0	0.1	0.38	0.88	0.53	328	538	44
**day**	**yes**	**0.5**	**1**	**0.57**	**0.73**	**0.75**	**0.74**	**278**	**102**	**94**
week*	yes	1	0	0.1	0.34	0.96	0.50	92	181	4
**week**	**yes**	**1**	**1**	**0.74**	**0.77**	**0.74**	**0.76**	**71**	**21**	**25**
dataset*	yes	1	0	0.1	0.25	1.00	0.40	23	70	0
**dataset**	**yes**	**1.5**	**0**	**0.8**	**0.90**	**0.78**	**0.84**	**18**	**2**	**5**

* Default BirdNET settings with week included. Bold are the best settings we recommend.

### Confirmation test

**Fig 3:**
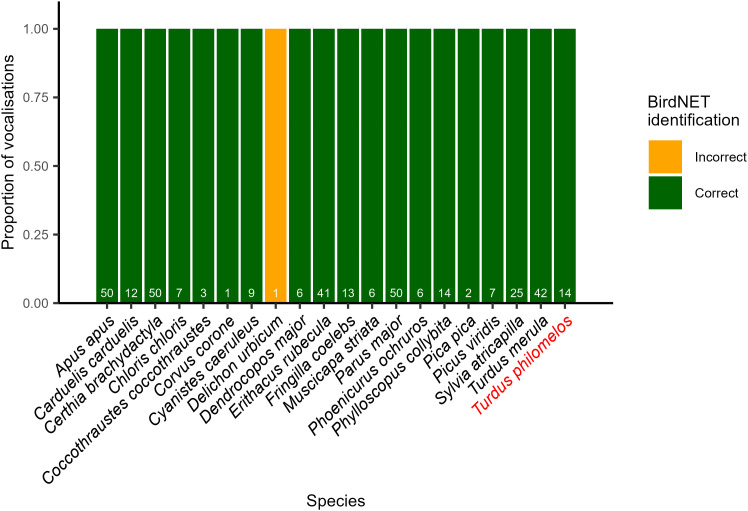
Accuracy of BirdNET species predictions based on manual checking. The proportion of BirdNET detections from the dataset level that were manually checked by an expert and determined to be correct or incorrect. A maximum of 50 random detections per species were checked. If a species was detected less than 50 times, all detections were checked. White numbers are the number of detections checked (if less than 50, only that number were available). The red label denotes the species missed in our expert identification that BirdNET identified.

In our confirmation test, conducted using the best parameter settings identified for data aggregated across the entire dataset, BirdNET detected 20 species. Among these were two species—*Delichon urbicum* (Western house martin) and *Turdus philomelos* (Song thrush)—that were not present in the expert identifications and were flagged as false positives. However, after manually reviewing the audio clips associated with these detections, we confirmed that all 14 detections of Song thrush were, in fact, correct. Only the single detection of Western house martin remained a true false positive (Fig 3). With these adjustments, the F1 score for the full-dataset level was revised to 0.86 (Precision = 0.95, Recall = 0.792). The expert identification included an additional five species missed by BirdNET. It is important to note that these false negatives were not addressed in the confirmation test, as they represent species that were not detected by the model. In our check of the false negative species, we found that their confidence scores ranged from a max of 0.37 to a max of 0.68 (Table S5 in S1 Text). As such, a lower minimum confidence threshold would have included some of these species, but at the cost of additional false positives. Lowering the minimum confidence to 0.54, for example, reduced the false negatives to 2, but at the cost of an additional nine false positive species, which would result in an overall lower F1 score.

## Discussion

Our study adds to the growing body of work evaluating the performance of BirdNET and is, to our knowledge, currently one of the few to systematically investigate the effect of varying BirdNET parameters. While current acoustic monitoring practices recommend short recordings during peak activity periods [13,18], BirdNET eliminates the need to listen to entire recordings, allowing for longer and more frequent data collection. As highlighted by our temporal aggregation, BirdNET performs better when aggregating results over longer periods, which also enhances monitoring by capturing species with varying activity patterns [19]. As with longer recordings [11,20,21], aggregating multiple shorter recordings gives BirdNET more opportunities to correctly detect a species with high confidence. However, BirdNET’s default settings produced substantial overdetection (13,787 detections from 93 species versus expert identification of 9,466 vocalisations from 23 species), highlighting the importance of parameter optimisation. Adjusting the parameters, more specifically, by including the week of the year, increasing the overlap to one or two seconds, and using a higher than default confidence threshold produced better results, especially when using short recordings as we did here. When provided with the correct settings and sufficient data (e.g., aggregated over longer periods or longer recordings), BirdNET can approach the performance of an expert and, in our case, even detected a species missed by our experts.

While even with our best result, BirdNET missed five species identified by the experts, this comparison should be considered in the context of how expert data are typically collected in practice. Our expert annotations were based on the same recordings analysed by BirdNET, whereas field-based monitoring commonly relies on point counts, which have well-documented limitations. Point counts are subject to observer bias, constrained by limited duration [22], and studies have shown variable performance compared to acoustic monitoring methods, with some finding acoustic surveys equivalent to point counts [22] while others demonstrate better detection rates for acoustic approaches [23,24]. Given these considerations, our acoustic-based expert annotations may represent a higher standard than traditional point counts, suggesting that BirdNET’s performance could be more competitive with conventional field monitoring than our results initially indicate. However, the relative performance of these approaches likely depends on specific research goals and the temporal resolution of interest [23,24]. Recent comparative studies support this conclusion, showing BirdNET performs favourably against point count methods when longer recordings are used [21,25]. As such, our results support that BirdNET can be used to monitor birds, even in acoustically complex environments such as cities.

Based on our findings across different temporal aggregations, we can deduce some generalisations for running BirdNET. Since our best-performing parameter settings always included the week of the year, we recommend general monitoring to include the week of the year and location. It should be noted that using a custom species list for your region and time of year can provide a similar effect as week of the year, which filters species occurrences based on eBird data. Overlap had a more significant impact on performance than sensitivity. We recommend using an overlap between one and two seconds for short one to five-minute recording schemes. When aggregating over longer periods (or potentially when using longer recordings), an overlap may not be necessary, as vocalisations that may be missed or unidentifiable from being cut are likely to occur again. Default sensitivity (1.0) generally produced the same results as higher or lower sensitivities while maintaining higher minimum confidence thresholds. We, therefore, recommend using the default sensitivity. For short recordings, we recommend using a moderate minimum confidence threshold that balances true and false positive rates (Table 4). Our urban dataset suggested optimal performance around 0.54 or higher, but users should validate appropriate thresholds for their specific recording environments, as factors such as background noise, recording quality, and habitat type may influence the detection of a species [6,26]. For example, the European Robin was the most frequently detected species by BirdNET under default settings yet ranked only fifth in expert identifications. Notably, most of these detections had low confidence scores. While confidence thresholds can vary significantly per species [12], a sufficiently high universal threshold, as shown by our results, will ensure the detections for most species have a high probability of being true. When aggregating data over time (or likely when using longer recordings), a higher confidence threshold yields the most reliable results (Table 4), although such a high threshold may exclude some low confidence but valid detections.

**Table 4:** Recommended BirdNET parameter settings and optional validation/postprocessing based on recording characteristics and research objectives.

Parameter	Use for	Recommendation	Rationale
**1. Main parameters**			
**Week**	All recordings	Always include*	Best performance with this parameter across all temporal resolutions
**Location**	All recordings	Always include*	Essential for regional species filtering
**Sensitivity**	All recordings	1.0 (default)	Has only a marginal effect on BirdNET performance
**2. Recording/data-specific parameters**			
**Overlap**	Short recordings/ no aggregation	1-2 Seconds	Reduces the chance of missing vocalisations that may otherwise be cut off at segment boundaries
**Overlap**	Long recordings/ aggregating data over longer periods (e.g., several weeks)**	0 Seconds (default)	Longer recordings increase the likelihood of repeated and high-confidence detections. Using overlap adds processing time with little benefit
**3. Confidence threshold Selection**			
**Confidence**	Short recordings/ no aggregation	Moderate (e.g., ≥ 0.54)	Balances false negatives and false positives on short recordings
**Confidence**	Long recordings/ aggregating data over longer periods (e.g., several weeks)**	Higher threshold	Reduces false positives by only retaining higher-confidence detections, which are more likely to be true
**4. Optional additional validation and post-processing steps**			
	All applications	Remove singletons & validate species with ≤10 detections ***	Removes singletons and verifies rare detections, which are often false positives, improving agreement with expert identifications.
	Sites with prior ornithological data	Check unlikely species only	Efficiently removes false positive species by focusing validation on improbable species
	Creating species lists per site	Validate detections sorted by highest to lowest confidence per species until a true positive is found	A single confirmed detection is sufficient to establish species presence
	Activity studies/ all applications	Calculate individual species thresholds	Retains more detections while reducing false positives, and may better reflect activity

* Custom species lists for specific regions and time periods can provide similar benefits to week-of-year filtering through eBird-based occurrence data.

** Aggregating over longer periods provides similar benefits to longer recordings, namely, it increases the likelihood of high-confidence detections occurring.

*** Our results showed that setting a lower confidence threshold than our best settings revealed, removing singletons, and validating those species detected fewer than 10 times significantly improved agreement with the expert results.

We recommend that the results of BirdNET be validated, especially when using very short recordings (Table 4). While research goals will dictate the amount of validation necessary, we recommend a few quick methods to ensure the best results. If the researcher is familiar with what is likely to occur on their study site, only manually checking unlikely or uncommon species is likely to suffice. If confirming the presence of the species at a location (i.e., producing a species list) is the research goal, validation can be done easily by manually checking the top results for each species/site, as only one valid detection is needed to confirm occurrence [9]. Additionally, removing singletons or doubletons and checking only infrequently detected species is likely to provide more accurate species lists than just the raw output of BirdNET. As our confirmation test showed, had we lowered the confidence threshold to 0.54, removed the singletons and checked the species that were detected 10 or fewer times, we would have had a species list closer to that of the expert, missing only two species. This highlights how simple validation and filtering steps can significantly improve agreement between expert and automated methods.

We recognise that current practice recommends using individual species thresholds, as model performance can vary greatly between species [6,12]. We think it is important to let the research question dictate which method should be used. Creating individual species scores requires a significant upfront investment in time and requires expert knowledge of the different vocalisations a species can make. Further, the creation of species-specific thresholds assumes that a dataset contains enough detections across the confidence range, although a universal number of detections has yet to be determined. Research questions for which the number of valid vocalisations is important, e.g., studies investigating activity patterns [10], could benefit from species-specific thresholds as they maintain a greater number of detections. For example, Tseng et al. (2025) [12] found that using individual species scores retained a much larger number of detections (70 ± 37%) than a universal threshold (17 ± 14%), as we use here. Still, it has yet to be determined if these thresholds are transferable across time (e.g., season, time of day, years) and space (e.g., regions and habitats). Until standardised species-specific thresholds become available, for short-term studies or rapid biodiversity assessments, universal thresholds with simple validation procedures may be sufficient and more resource efficient. Therefore, it is important to consider the aims of a project when deciding if a universal threshold is adequate or if individual thresholds should be calculated.

Our study provides additional support for BirdNET as a practical tool for species identification, particularly in urban environments, but also highlights some limitations that require careful consideration. We show that appropriate recording strategies and utilising or adjusting key parameters—such as including week of the year, increasing overlap for short recordings, and using a higher minimum confidence threshold—can substantially improve detection performance. It should be noted that our results represent a single site in a southern German city, and results from different regions or environments may vary. While we acknowledge that the universal confidence thresholds we propose may not suit all research contexts, with basic validation or filtering (e.g., checking uncommon or infrequent species), they can still yield ecologically useful results. The universal threshold approach offers reliable presence-absence data in a fast and efficient way as long as species-specific thresholds are not readily available. BirdNET effectively overcomes some of the limitations of conventional ornithological sampling methods, thus positioning it as a valuable asset in the ongoing quest for comprehensive and efficient biodiversity monitoring practices, offering new research opportunities in ecology and ornithology.

## Supporting information

S1 Text Supplementary tables of BirdNET and expert comparison analyses. This supplement includes detailed ANOVA results, species-level identification summaries by experts and BirdNET, F1 score statistics across parameter combinations, and confidence range for false negatives under optimal settings at dataset aggregation.
(DOCX)
