# Supplementary material for: BirdNET can be as good as experts for acoustic bird monitoring in a European city
Source: PLoS One. 2025 Sep 11;20(9):e0330836. doi: 10.1371/journal.pone.0330836 (PMC12425287; doi:10.1371/journal.pone.0330836)
Supplement: S1 Text — This supplement includes detailed ANOVA results, species-level identification summaries by experts and BirdNET, F1 score statistics across parameter combinations, and confidence range for false negatives under optimal settings at dataset aggregation. (DOCX) [file pone.0330836.s001.docx]

Fairbairn et al., BirdNET can be as good as experts for acoustic bird monitoring in a European city

| Supplementary Table S1. ANOVA results from a linear mixed effects model for F1 score and the BirdNET parameters sensitivity, overlap, and minimum confidence plus four data aggregation levels. | |
| --- | --- |
| **variable** | **F and p value** |
| level | F_3,63_=147.21; p<0.001 |
| sensitivity | F_1,20_=5.11; p=0.035 |
| overlap | F_1,20_=9.83; p=0.005 |
| minconf^2^ | F_1,7674_=5023.03; p<0.001 |
| level:sensitivity | F_3,63_=35.17; p=0.001 |
| level:overlap | F_3,63_=19.56; p=0.001 |
| level:minconf^2^ | F_3,7674_=750.56; p<0.001 |
| sensitivity:overlap | F_1,20_=0.43; p=0.521 |
| sensitivity:minconf^2^ | F_1,7674_=239.59; p<0.001 |
| overlap:minconf^2^ | F_1,7674_=603.84; p<0.001 |

| Supplementary Table S2. Expertly identified species with the total number of times each species was identified in the data. | |
| --- | --- |
| **Species** | **n identifications** |
| *Certhia brachydactyla* | 2894 |
| *Turdus merula* | 1078 |
| *Apus apus* | 817 |
| *Parus major* | 798 |
| *Sylvia atricapilla* | 555 |
| *Erithacus rubecula* | 527 |
| *Corvus corone* | 458 |
| *Phylloscopus collybita* | 401 |
| *Cyanistes caeruleus* | 380 |
| *Fringilla coelebs* | 323 |
| *Chloris chloris* | 321 |
| *Muscicapa striata* | 263 |
| *Phoenicurus ochruros* | 179 |
| *Passer montanus* | 136 |
| *Carduelis carduelis* | 118 |
| *Dendrocopos major* | 82 |
| *Coccothraustes coccothraustes* | 40 |
| *Picus viridis* | 28 |
| *Sitta europaea* | 26 |
| *Columba palumbus* | 15 |
| *Regulus regulus* | 12 |
| *Pica pica* | 8 |
| *Streptopelia decaocto* | 7 |
| **Total** | 9466 |

| Supplementary Table S3. Species identified by BirdNET (default settings, minimum confidence 0.1) with the total number of times each species was identified. | |
| --- | --- |
| **Species** | **n identifications** |
| *Erithacus rubecula* | 2890 |
| *Turdus merula* | 1820 |
| *Parus major* | 1264 |
| *Certhia brachydactyla* | 1037 |
| *Apus apus* | 893 |
| *Turdus philomelos* | 868 |
| *Corvus corone* | 622 |
| *Sylvia atricapilla* | 532 |
| *Cyanistes caeruleus* | 462 |
| *Phylloscopus collybita* | 326 |
| *Carduelis carduelis* | 292 |
| *Chloris chloris* | 267 |
| *Coccothraustes coccothraustes* | 245 |
| *Fringilla coelebs* | 242 |
| *Passer domesticus* | 242 |
| *Anthus trivialis* | 207 |
| *Muscicapa striata* | 196 |
| *Phoenicurus ochruros* | 163 |
| *Corvus cornix* | 152 |
| *Dendrocopos major* | 122 |
| *Delichon urbicum* | 104 |
| *Motacilla cinerea* | 77 |
| *Motacilla alba* | 52 |
| *Actitis hypoleucos* | 48 |
| *Fulica atra* | 48 |
| *Regulus regulus* | 44 |
| *Turdus viscivorus* | 36 |
| *Picus viridis* | 29 |
| *Columba palumbus* | 28 |
| *Gallinula chloropus* | 24 |
| *Chroicocephalus ridibundus* | 23 |
| *Emberiza schoeniclus* | 23 |
| *Prunella modularis* | 23 |
| *Periparus ater* | 21 |
| *Aegithalos caudatus* | 19 |
| *Regulus ignicapilla* | 19 |
| *Charadrius dubius* | 18 |
| *Corvus frugilegus* | 18 |
| *Alauda arvensis* | 17 |
| *Pica pica* | 17 |
| *Emberiza citrinella* | 16 |
| *Troglodytes troglodytes* | 16 |
| *Ficedula hypoleuca* | 15 |
| *Motacilla flava* | 15 |
| *Poecile palustris* | 15 |
| *Anas platyrhynchos* | 14 |
| *Anas crecca* | 12 |
| *Turdus pilaris* | 11 |
| *Ardea cinerea* | 10 |
| *Linaria cannabina* | 10 |
| *Buteo buteo* | 8 |
| *Alcedo atthis* | 6 |
| *Anthus pratensis* | 5 |
| *Corvus corax* | 5 |
| *Dryocopus martius* | 5 |
| *Emberiza calandra* | 5 |
| *Passer montanus* | 5 |
| *Sitta europaea* | 5 |
| *Falco tinnunculus* | 4 |
| *Grus grus* | 4 |
| *Phoenicurus phoenicurus* | 4 |
| *Pyrrhula pyrrhula* | 4 |
| *Rallus aquaticus* | 4 |
| *Serinus serinus* | 4 |
| *Spinus spinus* | 4 |
| *Columba livia* | 3 |
| *Mareca strepera* | 3 |
| *Oriolus oriolus* | 3 |
| *Saxicola rubetra* | 3 |
| *Saxicola rubicola* | 3 |
| *Streptopelia decaocto* | 3 |
| *Alopochen aegyptiaca* | 2 |
| *Corvus monedula* | 2 |
| *Fringilla montifringilla* | 2 |
| *Hirundo rustica* | 2 |
| *Lophophanes cristatus* | 2 |
| *Loxia curvirostra* | 2 |
| *Milvus milvus* | 2 |
| *Poecile montanus* | 2 |
| *Sturnus vulgaris* | 2 |
| *Sylvia borin* | 2 |
| *Tringa nebularia* | 2 |
| *Anser anser* | 1 |
| *Bucephala clangula* | 1 |
| *Certhia familiaris* | 1 |
| *Circus aeruginosus* | 1 |
| *Curruca communis* | 1 |
| *Curruca curruca* | 1 |
| *Luscinia megarhynchos* | 1 |
| *Sterna hirundo* | 1 |
| *Tachybaptus ruficollis* | 1 |
| *Tringa ochropus* | 1 |
| *Vanellus vanellus* | 1 |
| **Total** | 13787 |

| **Supplementary Table S4**. Summary statistics of F1 score for different parameter combinations, with each row showing statistics across the full range of minimum confidence thresholds (0.1-1). F1 scores represent variation across all confidence thresholds for each combination of aggregation level, sensitivity, and overlap parameter settings. | | | | | | | | |
| --- | --- | --- | --- | --- | --- | --- | --- | --- |
| level | week | overlap | sensitivity | min_f1 | max_f1 | mean_f1 | median_f1 | sd_f1 |
| minute | no | 0 | 0.5 | 0.41 | 0.534 | 0.506 | 0.519 | 0.032 |
| minute | no | 0 | 1 | 0.329 | 0.534 | 0.479 | 0.499 | 0.056 |
| minute | no | 0 | 1.5 | 0.124 | 0.534 | 0.388 | 0.406 | 0.121 |
| minute | no | 1 | 0.5 | 0.442 | 0.552 | 0.517 | 0.527 | 0.031 |
| minute | no | 1 | 1 | 0.344 | 0.552 | 0.487 | 0.506 | 0.058 |
| minute | no | 1 | 1.5 | 0.106 | 0.552 | 0.392 | 0.412 | 0.125 |
| minute | no | 2 | 0.5 | 0.393 | 0.562 | 0.515 | 0.527 | 0.043 |
| minute | no | 2 | 1 | 0.298 | 0.562 | 0.484 | 0.501 | 0.068 |
| minute | no | 2 | 1.5 | 0.088 | 0.562 | 0.39 | 0.411 | 0.132 |
| minute | no | 2.9 | 0.5 | 0.254 | 0.525 | 0.42 | 0.432 | 0.079 |
| minute | no | 2.9 | 1 | 0.18 | 0.524 | 0.406 | 0.432 | 0.105 |
| minute | no | 2.9 | 1.5 | 0.054 | 0.524 | 0.344 | 0.37 | 0.147 |
| minute | yes | 0 | 0.5 | 0.418 | 0.576 | 0.54 | 0.56 | 0.043 |
| minute | yes | 0 | 1 | 0.332 | 0.577 | 0.515 | 0.538 | 0.066 |
| minute | yes | 0 | 1.5 | 0.178 | 0.576 | 0.425 | 0.447 | 0.127 |
| minute | yes | 1 | 0.5 | 0.452 | 0.597 | 0.562 | 0.578 | 0.038 |
| minute | yes | 1 | 1 | 0.365 | 0.597 | 0.532 | 0.555 | 0.063 |
| minute | yes | 1 | 1.5 | 0.162 | 0.597 | 0.436 | 0.456 | 0.129 |
| minute | yes | 2 | 0.5 | 0.483 | 0.62 | 0.575 | 0.584 | 0.036 |
| minute | yes | 2 | 1 | 0.387 | 0.62 | 0.542 | 0.558 | 0.063 |
| minute | yes | 2 | 1.5 | 0.137 | 0.62 | 0.44 | 0.456 | 0.135 |
| minute | yes | 2.9 | 0.5 | 0.346 | 0.591 | 0.51 | 0.528 | 0.071 |
| minute | yes | 2.9 | 1 | 0.258 | 0.591 | 0.489 | 0.523 | 0.096 |
| minute | yes | 2.9 | 1.5 | 0.093 | 0.591 | 0.409 | 0.437 | 0.15 |
| day | no | 0 | 0.5 | 0.513 | 0.655 | 0.615 | 0.624 | 0.034 |
| day | no | 0 | 1 | 0.41 | 0.655 | 0.586 | 0.599 | 0.06 |
| day | no | 0 | 1.5 | 0.183 | 0.654 | 0.486 | 0.515 | 0.136 |
| day | no | 1 | 0.5 | 0.483 | 0.671 | 0.618 | 0.634 | 0.046 |
| day | no | 1 | 1 | 0.375 | 0.668 | 0.586 | 0.605 | 0.074 |
| day | no | 1 | 1.5 | 0.168 | 0.667 | 0.483 | 0.501 | 0.145 |
| day | no | 2 | 0.5 | 0.423 | 0.663 | 0.594 | 0.621 | 0.068 |
| day | no | 2 | 1 | 0.325 | 0.662 | 0.567 | 0.594 | 0.094 |
| day | no | 2 | 1.5 | 0.155 | 0.661 | 0.474 | 0.502 | 0.153 |
| day | no | 2.9 | 0.5 | 0.279 | 0.638 | 0.462 | 0.462 | 0.099 |
| day | no | 2.9 | 1 | 0.218 | 0.639 | 0.456 | 0.462 | 0.13 |
| day | no | 2.9 | 1.5 | 0.129 | 0.634 | 0.412 | 0.443 | 0.167 |
| day | yes | 0 | 0.5 | 0.613 | 0.707 | 0.676 | 0.681 | 0.025 |
| day | yes | 0 | 1 | 0.503 | 0.707 | 0.649 | 0.666 | 0.052 |
| day | yes | 0 | 1.5 | 0.277 | 0.705 | 0.55 | 0.588 | 0.13 |
| day | yes | 1 | 0.5 | 0.602 | 0.739 | 0.694 | 0.7 | 0.033 |
| day | yes | 1 | 1 | 0.507 | 0.738 | 0.663 | 0.684 | 0.061 |
| day | yes | 1 | 1.5 | 0.289 | 0.736 | 0.558 | 0.578 | 0.137 |
| day | yes | 2 | 0.5 | 0.544 | 0.738 | 0.685 | 0.704 | 0.05 |
| day | yes | 2 | 1 | 0.453 | 0.738 | 0.655 | 0.681 | 0.075 |
| day | yes | 2 | 1.5 | 0.273 | 0.738 | 0.556 | 0.577 | 0.14 |
| day | yes | 2.9 | 0.5 | 0.41 | 0.719 | 0.582 | 0.59 | 0.084 |
| day | yes | 2.9 | 1 | 0.347 | 0.721 | 0.571 | 0.59 | 0.111 |
| day | yes | 2.9 | 1.5 | 0.244 | 0.717 | 0.513 | 0.528 | 0.149 |
| week | no | 0 | 0.5 | 0.43 | 0.687 | 0.579 | 0.595 | 0.077 |
| week | no | 0 | 1 | 0.348 | 0.687 | 0.563 | 0.595 | 0.098 |
| week | no | 0 | 1.5 | 0.203 | 0.68 | 0.507 | 0.563 | 0.145 |
| week | no | 1 | 0.5 | 0.393 | 0.686 | 0.567 | 0.57 | 0.082 |
| week | no | 1 | 1 | 0.312 | 0.686 | 0.55 | 0.57 | 0.107 |
| week | no | 1 | 1.5 | 0.193 | 0.686 | 0.492 | 0.542 | 0.151 |
| week | no | 2 | 0.5 | 0.356 | 0.664 | 0.516 | 0.528 | 0.082 |
| week | no | 2 | 1 | 0.292 | 0.673 | 0.509 | 0.528 | 0.11 |
| week | no | 2 | 1.5 | 0.184 | 0.667 | 0.47 | 0.528 | 0.153 |
| week | no | 2.9 | 0.5 | 0.267 | 0.574 | 0.389 | 0.372 | 0.079 |
| week | no | 2.9 | 1 | 0.232 | 0.615 | 0.397 | 0.372 | 0.111 |
| week | no | 2.9 | 1.5 | 0.168 | 0.629 | 0.401 | 0.372 | 0.16 |
| week | yes | 0 | 0.5 | 0.569 | 0.737 | 0.675 | 0.694 | 0.049 |
| week | yes | 0 | 1 | 0.499 | 0.737 | 0.657 | 0.679 | 0.065 |
| week | yes | 0 | 1.5 | 0.365 | 0.73 | 0.6 | 0.625 | 0.105 |
| week | yes | 1 | 0.5 | 0.548 | 0.755 | 0.685 | 0.69 | 0.053 |
| week | yes | 1 | 1 | 0.475 | 0.755 | 0.665 | 0.678 | 0.074 |
| week | yes | 1 | 1.5 | 0.351 | 0.755 | 0.601 | 0.632 | 0.115 |
| week | yes | 2 | 0.5 | 0.504 | 0.742 | 0.644 | 0.661 | 0.061 |
| week | yes | 2 | 1 | 0.454 | 0.737 | 0.631 | 0.661 | 0.082 |
| week | yes | 2 | 1.5 | 0.345 | 0.736 | 0.584 | 0.621 | 0.116 |
| week | yes | 2.9 | 0.5 | 0.427 | 0.696 | 0.54 | 0.535 | 0.065 |
| week | yes | 2.9 | 1 | 0.396 | 0.718 | 0.545 | 0.535 | 0.094 |
| week | yes | 2.9 | 1.5 | 0.326 | 0.718 | 0.534 | 0.535 | 0.128 |
| dataset | no | 0 | 0.5 | 0.329 | 0.667 | 0.48 | 0.472 | 0.103 |
| dataset | no | 0 | 1 | 0.284 | 0.667 | 0.481 | 0.472 | 0.128 |
| dataset | no | 0 | 1.5 | 0.196 | 0.791 | 0.486 | 0.472 | 0.194 |
| dataset | no | 1 | 0.5 | 0.307 | 0.645 | 0.467 | 0.454 | 0.092 |
| dataset | no | 1 | 1 | 0.266 | 0.633 | 0.466 | 0.454 | 0.119 |
| dataset | no | 1 | 1.5 | 0.192 | 0.756 | 0.467 | 0.454 | 0.183 |
| dataset | no | 2 | 0.5 | 0.289 | 0.563 | 0.397 | 0.383 | 0.074 |
| dataset | no | 2 | 1 | 0.257 | 0.613 | 0.407 | 0.383 | 0.104 |
| dataset | no | 2 | 1.5 | 0.189 | 0.773 | 0.43 | 0.383 | 0.181 |
| dataset | no | 2.9 | 0.5 | 0.235 | 0.438 | 0.31 | 0.311 | 0.048 |
| dataset | no | 2.9 | 1 | 0.209 | 0.475 | 0.317 | 0.311 | 0.073 |
| dataset | no | 2.9 | 1.5 | 0.18 | 0.735 | 0.348 | 0.311 | 0.149 |
| dataset | yes | 0 | 0.5 | 0.451 | 0.745 | 0.596 | 0.6 | 0.09 |
| dataset | yes | 0 | 1 | 0.397 | 0.75 | 0.592 | 0.6 | 0.113 |
| dataset | yes | 0 | 1.5 | 0.319 | 0.837 | 0.589 | 0.6 | 0.171 |
| dataset | yes | 1 | 0.5 | 0.438 | 0.741 | 0.595 | 0.595 | 0.076 |
| dataset | yes | 1 | 1 | 0.387 | 0.717 | 0.587 | 0.595 | 0.101 |
| dataset | yes | 1 | 1.5 | 0.315 | 0.818 | 0.579 | 0.595 | 0.16 |
| dataset | yes | 2 | 0.5 | 0.407 | 0.667 | 0.524 | 0.53 | 0.065 |
| dataset | yes | 2 | 1 | 0.377 | 0.704 | 0.527 | 0.53 | 0.093 |
| dataset | yes | 2 | 1.5 | 0.309 | 0.829 | 0.544 | 0.53 | 0.165 |
| dataset | yes | 2.9 | 0.5 | 0.357 | 0.56 | 0.435 | 0.442 | 0.047 |
| dataset | yes | 2.9 | 1 | 0.333 | 0.594 | 0.441 | 0.442 | 0.072 |
| dataset | yes | 2.9 | 1.5 | 0.297 | 0.818 | 0.468 | 0.442 | 0.143 |

| **Supplementary Table S5.** Confidence range of false negative species from the best settings at the dataset aggregation. | |
| --- | --- |
| **Species** | **Confidence range** |
| Passer montanus | 0.1-0.42 |
| Sitta europaea | 0.1-0.69 |
| Columba palumbus | 0.1-0.59 |
| Regulus regulus | 0.1-0.55 |
| Streptopelia decaocto | 0.1-0.38 |
